# Within-game performance decrements in relation to peak fat oxidation, Fatmax and peak oxygen uptake in elite male footballers

**DOI:** 10.5114/biolsport.2026.158678

**Published:** 2026-03-04

**Authors:** Morten Bredsgaard Randers, Tue Rømer, Mikkel Thunestvedt Hansen, Christina Rohde Ruppert, Viktor Slott, Thomas Nielsen, Loftur Gísli Jóhannsson, Jesper Sangild, Jørn Wulff Helge, Peter Krustrup, Malte Nejst Larsen

**Affiliations:** 1 Department of Sports Science and Clinical Biomechanics, Sport and Health Sciences Cluster (SHSC), University of Southern Denmark, Odense, Denmark; 2 Department of Biomedical Sciences, Faculty of Health and Medical Sciences, University of Copenhagen, Copenhagen, Denmark; 3 Odense Boldklub, Odense, Denmark; 4 Danish Institute for Advanced Study (DIAS), University of Southern Denmark, Odense, Denmark; 5 Sport and Health Sciences, University of Exeter, Exeter, United Kingdom

**Keywords:** Physical match performance, Treadmill running, Maximal oxygen uptake, Fat oxidation capacity, High-intensity running

## Abstract

This study investigated whether fat oxidation capacity is associated with sustained physical performance during the final 30 minutes of elite football match play. Twenty-four professional male football players (age: 24.9 ± 4.0 years; $\dot{\text{V}}$O_2peak_: 59.1 ± 5.7 ml/min/kg) from the Danish Superliga completed laboratory testing to determine peak fat oxidation (PFO), Fatmax, and $\dot{\text{V}}$O_2peak_. From match tracking data, distances covered within speed zones, number of runs, and peak speed were analysed for full-match and changes between the first 60 and final 30 minutes. Correlations between laboratory and match variables were assessed. PFO was (mean ± SD) 0.48 ± 0.16 g/min (range: 0.24–0.78), Fatmax was 43 ± 10 %$\dot{\text{V}}$O_2peak_ (31–61), and $\dot{\text{V}}$O_2peak_ was 59.1 ± 5.7 ml/min/kg (49.7–71.3). Players covered 11,216 ± 789 m during a full match, including 899 ± 323 m > 20 km/h and performed 79 ± 29 high-intensity runs. PFO normalized to fat-free mass (PFO/FFM) was significantly correlated with attenuated declines in number of decelerations (r=0.42, P=0.042) in the final 30 minutes, whereas no other correlations with sustained physical performance variables were observed. $\dot{\text{V}}$O_2peak_ was associated with total distance (r=0.59, P=0.002), but not with performance decrements. Peak fat oxidation may play a modest but significant role in supporting repeated high-intensity decelerations late in games in male elite footballers. $\dot{\text{V}}$O_2peak_ was correlated to overall running performance but appears less informative for fatigue resistance.

## INTRODUCTION

Sustaining high performance during prolonged intermittent exercise requires efficient regulation of substrate utilization and energy supply. In sports like football, where matches last up to 90 or 120 minutes plus additional playing time, the balance between carbohydrate and fat metabolism may influence fatigue resistance and decision-making quality in critical late-game phases [1, 2]. Elite football imposes metabolic demands characterised by frequent high-intensity actions interspersed with prolonged periods of submaximal activity [3–5]. While decisive events are often preceded by intense actions such as sprints, accelerations, decelerations, and changes of direction [6, 7], 85–90% of match time is spent at low to moderate intensities where aerobic metabolism predominates [8]. During these periods, a greater capacity to oxidize fat may spare intramuscular glycogen and help delay fatigue and thereby sustain physical performance in the later stages of matches [1, 9, 10].

The concept of glycogen sparing through enhanced fat oxidation was established in the 1960s through foundational muscle biopsy studies [11, 12] and remains central to endurance exercise physiology [13–15]. While traditionally associated with continuous exercise, recent work suggests that fat oxidation may also support performance in intermittent sports like football [9, 16, 17].

Mechanistically, the decline in fat oxidation at higher exercise intensities is driven by both reduced fatty acid availability, impaired mitochondrial transport and a lower adenosine triphosphate (ATP) yield per oxygen when comparing fat to carbohydrate [18]. As intensity increases, adipose tissue blood flow decreases, limiting the mobilization of free fatty acids to the working muscles. Concurrently, intracellular factors such as carnitine depletion and exercise-induced acidosis inhibit the carnitine palmitoyltransferase I (CPT-I) enzyme, reducing the transport of long-chain fatty acids into the mitochondria [19]. Together, these limitations suppress the rate at which fatty acids can be oxidized, thereby shifting substrate reliance toward carbohydrate metabolism [20]. In football, where players alternate rapidly between intensities, those with low metabolic flexibility may experience higher glycogen depletion, impairing performance in the latter stages of matches [5, 8, 21]. This is particularly relevant given that depletion of intramyofibrillar glycogen has been linked to impaired sarcoplasmic reticulum calcium kinetics and reduced excitation-contraction coupling [22]. At a systemic level, increased concentrations of plasma free fatty acids and declining glycogen reserves have been documented during the final stages of football matches [1, 2, 10], suggesting that fat metabolism may play a critical role in maintaining physical capacity in the latter parts of matches.

Peak fat oxidation (PFO) and the relative exercise intensity at which it occurs (Fatmax) are gaining recognition as markers of metabolic flexibility [20]. PFO is modified by training, nutrition, and exercise modality [20], and football players have demonstrated some of the highest PFO values reported across athlete populations [9, 16, 17]. These findings challenge the assumption that fat metabolism is unimportant in football and suggest PFO may complement peak oxygen uptake ($\dot{\text{V}}$O_2peak_) in characterising team sport physical performance. A rather larger inter-individual variability highlights the importance of contextualizing PFO values within each athlete’s physiological profile [9, 17].

Nevertheless, the independent contribution of PFO to match performance remains debated. In a pre-season football study, Rømer, Hansen [17] observed concurrent increases in both PFO and $\dot{\text{V}}$O_2peak_ in sub-elite football players, but only $\dot{\text{V}}$O_2peak_ was associated with improved intermittent running performance (Yo-Yo IR2 performance). This divergence suggests that while PFO may be associated with a glycogen sparring effect, it may not directly predict high-intensity intermittent performance.

Taken together, these findings highlight a need to better understand the functional role of fat oxidation in football performance. Although $\dot{\text{V}}$O_2peak_ is a central determinant of aerobic capacity, PFO may provide additional insight into individual fatigue profiles and energy efficiency during match play. The current study therefore investigates whether higher fat oxidation capacity in elite male football players is associated physical match performance and within-match changes in performance reflecting fatigue in the latter part of matches.

## MATERIALS AND METHODS

### Design

A total of 58 professional male players from the Danish Superliga were recruited over three seasons (2021/22–2023/24). Players underwent a laboratory test battery, including whole-body dual-energy X-ray absorptiometry (DXA) scanning and a graded treadmill test to volitional exhaustion determine peak fat oxidation (PFO), Fatmax, and $\dot{\text{V}}$O_2peak_. Match performance data were extracted from Second Spectrum tracking during full 90-minute matches played 15 (range: 4–24) days from to the laboratory testing session. To be included in the study, players should have a data set including laboratory testing and at least one match, during which they participated in at least 80 minutes of match time, within 4 weeks from the laboratory testing. This criterion led to a total inclusion of 24 players. Characteristics of these players at the time of laboratory testing are presented in table 1. Prior to inclusion in the study all players provided informed consent. The study was approved by local ethics committee and was conducted in accordance with the Declaration of Helsinki.

**TABLE 1:** Anthropometrics and laboratory test results

**Age (years)**	24.9 ± 4.0
**Weight (kg)**	78.1 ± 6.3
**Height (cm)**	182.6 ± 6.1
**Fat free mass (kg)**	69.4 ± 5.9
**Fat mass (kg)**	8.7 ± 1.5
**Fat percentage (%)**	11.2 ± 1.9
**Fatmax (% of $\dot{\text{V}}$O_2peak_)**	42.8 ± 9.5
**PFO (g/min)**	0.48 ± 0.16
**PFO per FFM (mg/min/kg)**	6.9 ± 2.4
**$\dot{\text{V}}$O_2peak_ (ml/min)**	4615 ± 574
**$\dot{\text{V}}$O_2peak_ (ml/min/kg)**	59.1 ± 5.7

Data are presented as mean ± SD. PFO: Peak fat oxidation, FFM: Fat free mass, $\dot{\text{V}}$O_2peak_: Peak oxygen uptake.

### Laboratory Testing Procedures

All laboratory assessments were conducted at a facility located near the football club. Participants were instructed to arrive in a fasted state. Actual fasting durations ranged from 4 to 13 hours, with a mean of 6 hours and 20 minutes. Moreover, players were instructed to refrain from strenuous physical activity (36 hours), caffeine and nutritional supplements (12 hours) hours prior to testing. Upon arrival, anthropometric measurements were recorded. Height was measured (Tanita Leicester Stadiometer), and body weight and composition were assessed while players wore minimal clothing using two methods: a multifrequency bioelectrical impedance analyser (InBody 270, Biospace, San Diego, CA) and dual-energy X-ray absorptiometry (DXA; Lunar iDXA, GE Healthcare, UK).

### Treadmill test

After body composition assessments, participants were briefed on the exercise protocol. A graded treadmill test was used to determine peak fat oxidation (PFO) and Fatmax. Testing was conducted on a motorized treadmill (Woodway Pro XL, Woodway Inc., USA or Rodby RL2000E, Rodby, Sweden), with simultaneous measurements of pulmonary gas exchange rates using a mixing chamber system (Quark CPET, Cosmed, Rome, Italy).

The protocol was adapted from Rømer, Hansen [17]. In brief, gas and flow calibration were performed prior to testing. Participants were fitted with a heart rate monitor and a headgear system (Hans-Rudolph Inc., Kansas City, MO), which connected a rubber face mask to a two-way non-rebreathing valve and a hose transferring expired air to the mixing chamber. The protocol began with a 5-minute seated rest period, during which participants were instructed to breathe normally. This was followed by a 5-minute warm-up at 6 km/h and 1% incline. Subsequently, treadmill speed increased to 8 km/h for 3 minutes, and then by 2 km/h every 3 minutes while maintaining a 1% incline. The graded phase continued until a respiratory exchange ratio (RER) > 0.95 was reached, marking the transition from Fatmax to $\dot{\text{V}}$O_2peak_ testing.

During the $\dot{\text{V}}$O_2peak_ phase, speed was initially increased by 1 km/h every minute until reaching 16 km/h. Thereafter, incline increased by 1% every minute until 4% was reached, after which treadmill speed was increased by 1 km/h per minute while keeping the incline constant at 4%. Participants were strongly encouraged to continue until exhaustion, at which point the test was terminated. Immediately following test completion, the mask was removed and perceived exertion was rated using the Borg scale (6–20) [23].

### Pulmonary gas exchange and Data Analysis

Pulmonary gas exchange data were recorded in 10-second sampling intervals and processed as 30-second rolling averages using Omnia software (Cosmed, Rome, Italy). Fat oxidation rates (g/min) were calculated using the stoichiometric equation proposed by [24], under the assumption of negligible protein oxidation:

$$\text{Fat oxidation }(\text{g}/\min)=1.67*{\dot{\text{V}}\text{O}}_{2}-1.67*{\dot{\text{V}}\text{O}}_{2}$$

For each player, gas exchange data were manually reviewed. The average $\dot{\text{V}}$O_2_ and $\dot{\text{V}}$CO_2_ over a 60-second steady-state period was calculated for each stage, excluding the first 90 seconds and the final 30 seconds. For the resting and warm-up periods, the first 210 seconds and the last 30 seconds were excluded. Fat oxidation and relative intensity (%$\dot{\text{V}}$O_2peak_) were calculated for each stage, and individual PFO and Fatmax values were determined using a thirddegree polynomial regression of fat oxidation as a function of exercise intensity.

$\dot{\text{V}}$O_2peak_ was defined as the highest 30-second average $\dot{\text{V}}$O_2_ value obtained during the test. A $\dot{\text{V}}$O_2peak_ value was accepted if a plateau was observed (i.e., an increase of < 2 mL · min^−1^ · kg^−1^ with increased workload), or if two of the following secondary criteria were met: a) RPE > 17 on the Borg scale [23]; b) RER > 1.10; c) heart rate > 95% of age-predicted maximum, calculated as ± 5 BMP from 220 – age). A $\dot{\text{V}}$O_2_ plateau was observed in 18 of 24 participants and of the remaining six players, five met at least two of the secondary criteria.

### Match analysis

Physical match performance data was extracted from the Second Spectrum platform in 1-min intervals. Physical match performance variables were calculated across the entire match. To evaluate withinmatch decrements variables were calculated per minute for two time periods: the last 30 minutes (60–90 min) relative to the first 60 minutes (0–60 min). The included variables were distance covered in total and within speed zones 0–7 km/h, 7–15 km/h, 15–20 km/h, 20–25 km/h, and > 25 km/h, as well as distance > 15 km/h and > 20 km/h. Number of runs > 20 km/h, 20–25 km/h and > 25 km/h were also collected, and number of accelerations (> 3 m/s^2^) and decelerations (< -3 m/s^2^) as well as top speed during match periods were assessed.

### Statistical analysis

In cases where multiple matches were available within the timeframe surrounding the laboratory testing, the match with the highest highintensity distance (> 20 km/h) covered during the entire match was included in the statistical analysis.

Statistical analysis was performed in R Studio (ver. 2025.05.1 build 51. All data were checked for normal distribution using Shapiro-Wilk test. Normal distributed data were analysed using Correlations between physical test data and match performance changes were assessed using Pearson’s correlation coefficients for normally distributed data and Spearman’s for non-normally distributed data. Distance sprinting and Fatmax were in general not normally distributed, whereas number of accelerations were not normally distributed for changes within the match (table 3), whereas number of decelerations were not normally distributed for whole match data (table 4). A significance threshold of P < 0.05 was applied. Descriptive data are presented as means ± standard deviation.

## RESULTS

### Laboratory tests

Laboratory test results are presented in Table 1. In brief, PFO ranged 0.24 to 0.78 g/min, corresponding to 3.6 to 12.6 mg/min/kg fat-free mass. Fatmax occurred at 31 to 61 % of $\dot{\text{V}}$O_2peak_. Absolute $\dot{\text{V}}$O_2peak_ ranged from 3.48 to 5.67 l/min, corresponding to a relative $\dot{\text{V}}$O_2peak_ value of 49.7 to 71.3 ml/min/kg.

### Match performance

Match performance data for the entire match as well as changes in match performance in the last 30 min (60–90 min) relative to performance during the first 60 min (0–60 min) are presented in Table 2. Briefly, playing time varied from 87 to 104 minutes, during which players covered between 9,647 and 12,832 m. Of this, 1,789 to 3,644 m were performed at speeds > 15 km/h, and 455 to 1503 m > 20 km/h. The number of high-intensity runs (> 20 km/h) was 29 to 136, with 34 to 119 accelerations > 3 m/s^2^ and 56 to 139 decelerations a> 3 m/s^2^. Peak in-game running speed ranged from 26.9 to 34.7 km/h. When comparing performance in the final 30 minutes (60–90 min) with the first 60 minutes of match play, a general decline was observed across most physical performance variables (Table 2). These decrements included total distance, moderate and high-speed running, as well as number of high-intensity actions, accelerations and decelerations. In contrast, distance covered by sprinting as well as number of sprints showed large variability and, on average, increased relative to the first 60 minutes.

**TABLE 2:** Physical match performance during the entire match and performance in 60–90 min relative to 0–60 min

	Performance total match	Performance 60–90 min relative to 0–60 min
Playing time (min)	100.2 ± 4.7	
Total distance (m)	11216 ± 789	92 ± 7%
Distance walking (0–7 km/h) (m)	3279 ± 277	101 ± 7%
Distance jogging (7–15 km/h) (m)	5265 ± 570	88 ± 11%
Distance running at moderate speed (15–20 km/h) (m)	1774 ± 287	87 ± 17%
Distance running at high speed (20–25 km/h) (m)	677 ± 215	88 ± 33%
Distance sprinting (> 25 km/h) (m)	222 ± 131	197 ± 325%
Distance running (> 15 km/h) (m)	2673 ± 459	87 ± 15%
Distance at high intensity (> 20 km/h) (m)	899 ± 323	91 ± 31%
Number of high-speed runs (20–25 km/h) (n)	65 ± 22	83 ± 23%
Number of sprints (> 25 km/h) (n)	14 ± 8	105 ± 97%
Number of high-intensity runs (> 20 km/h) (n)	79 ± 29	85 ± 24%
Number of accelerations (> 3 m/s^2^) (n)	86 ± 19	93 ± 24%
Number of decelerations (> 3 m/s^2^) (n)	111 ± 21	88 ± 24%
Top speed (km/h)	31.2 ± 2.2	94 ± 7%

Data are presented as mean ± SD.

### Correlations between laboratory tests variables and match performance metrics

**FIG. 1:**
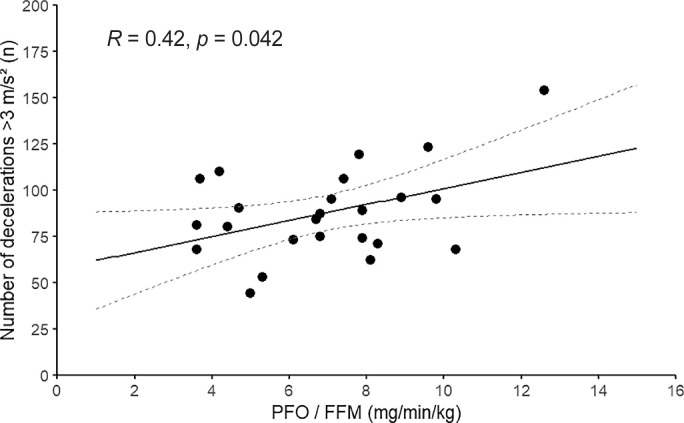
Pearson correlation between peak fat oxidation (PFO) per fat free mass (FFM) vs. decrement in number of decelerations > -3 m/s^2^. Individual data, regression line and confidence intervals are shown.

**FIG. 2:**
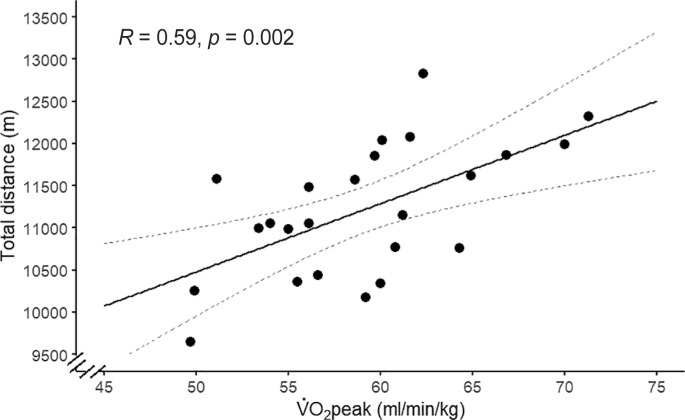
Pearson correlation between $\dot{\text{V}}$O_2peak_ (ml/min/kg) vs. total distance covered. Individual data, regression line and confidence intervals are shown.

Correlations between laboratory test measures and match performance metrics are summarized in Tables 3 and 4. No significant associations were observed between PFO or Fatmax and any of the match performance metrics. However, PFO normalized to fat-free mass showed significant positive correlations with the ability to sustain the number of decelerations (r = 0.42, P = 0.042; Figure 1) in the final 30 minutes of match play. A similar trend was observed for absolute PFO, for which the number of accelerations also tended to be correlated, but these correlations did not reach statistical significance. Relative $\dot{\text{V}}$O_2peak_ was significantly associated with the ability to maintain jogging distance (7–15 km/h) during the final 30 minutes (r = 0.50, P = 0.013), but not with any other match decrement variables. Conversely, when assessing total match performance (Table 4), $\dot{\text{V}}$O_2peak_ was significantly correlated with total distance (r = 0.59, P = 0.002; Figure 2), as well as jogging and running at moderate speed (r = 0.44–0.48, P < 0.05). Moreover, $\dot{\text{V}}$O_2peak_ was correlated with distance covered > 15 km/h and number of decelerations. No significant correlations were observed between any test variables and sprint distance or top speed, neither in full-match data nor in performance decrement variables.

**TABLE 3:** Correlations between laboratory tests variable and decrements in match performance.

	PFO (g/min)		PFO/FFM (mg/min/kg)		Fatmax (% of $\dot{\text{V}}$O_2peak_)		$\dot{\text{V}}$O_2peak_ (ml/min/kg)	

	r	P	r	P	r	P	r	P
Total distance	−0.18	0.396	−0.20	0.350	0.18	0.413	0.30	0.148
Distance walking (0–7 km/h)	−0.16	0.463	−0.18	0.408	0.04	0.870	−0.13	0.557
Distance jogging (7–15 km/h)	−0.13	0.543	−0.16	0.462	−0.01	0.963	**0.50**	**0.013**
Distance running at moderate speed (15–20 km/h)	−0.19	0.379	−0.20	0.344	0.17	0.422	−0.05	0.829
Distance running at high speed (20–25 km/h)	0.00	0.994	−0.02	0.935	0.32	0.127	0.04	0.842
Distance sprinting (> 25 km/h)	0.02	0.924	−0.03	0.898	−0.20	0.395	−0.09	0.691
Distance running (> 15 km/h)	−0.09	0.689	−0.09	0.681	0.21	0.322	−0.07	0.761
Distance at high intensity (> 20 km/h)	0.05	0.824	0.04	0.845	0.19	0.399	0.01	0.947
Number of high-speed runs (20–25 km/h)	0.18	0.398	0.20	0.344	0.13	0.552	0.10	0.632
Number of sprints (> 25 km/h)	0.26	0.253	0.16	0.493	−0.13	0.564	−0.04	0.862
Number of high-intensity runs (> 20 km/h)	0.19	0.385	0.21	0.332	0.08	0.694	0.10	0.631
Number of accelerations (> 3 m/s^2^)	*0.34*	*0.094*	0.31	0.146	−0.16	0.452	0.01	0.958
Number of decelerations (> 3 m/s^2^)	*0.40*	*0.053*	**0.42**	**0.042**	−0.07	0.751	−0.18	0.403
Top speed	0.13	0.546	0.11	0.622	0.24	0.263	0.07	0.748

All data were checked for normality using Shapiro-Wilk’s test. Normal distributed data: Pearsson’s correlation (not underlined); Spearman’s correlation (underlined). Bold denotes significant correlations (P < 0.05). Italic denotes 0.10 > P > 0.05. PFO: Peak fat oxidation, FFM: Fat free mass, $\dot{\text{V}}$O_2peak_: Peak oxygen uptake.

**TABLE 4:** Correlations between laboratory tests variables and total match performance.

	PFO (g/min)		PFO/FFM (mg/min/kg)		Fatmax (% of $\dot{\text{V}}$O_2peak_)		$\dot{\text{V}}$O_2peak_ (ml/min/kg)	

	r	P	r	P	r	P	r	P
Total Distance	0.05	0.808	0.06	0.790	−0.01	0.955	**0.59**	**0.002**
Distance Walking (0–7 km/h)	0.27	0.208	*0.37*	*0.077*	−0.15	0.481	0.10	0.649
Distance Jogging (7–15 km/h)	−0.21	0.324	−0.26	0.214	−0.16	0.443	**0.44**	**0.031**
Distance Running Below High Speed (15–20 km/h)	0.09	0.692	0.05	0.810	0.08	0.693	**0.48**	**0.017**
Distance Running at High Speed (20–25 km/h)	0.27	0.211	0.29	0.164	0.17	0.418	0.21	0.312
Distance Sprinting (> 25 km/h)	0.17	0.425	0.23	0.275	0.28	0.185	−0.02	0.921
Distance Running (> 15 km/h)	0.19	0.373	0.20	0.341	0.18	0.412	**0.41**	**0.045**
Distance at High-Intensity (> 20 km/h)	0.19	0.361	0.24	0.253	0.23	0.287	0.16	0.454
Number of Non-Sprint High-Speed Runs (20–25 km/h)	0.13	0.537	0.18	0.408	0.21	0.334	0.09	0.673
Number of sprints (> 25 km/h)	0.13	0.547	0.17	0.423	*0.36*	*0.087*	−0.03	0.886
Number of High-Intensity Runs (> 20 km/h)	0.13	0.535	0.18	0.406	0.22	0.308	0.06	0.784
Number of accelerations (> 3 m/s^2^)	−0.30	0.148	−0.25	0.243	−0.30	0.154	0.14	0.526
Number of decelerations (> 3 m/s^2^)	−0.13	0.535	−0.16	0.429	**−0.51**	**0.011**	**0.44**	**0.030**
Top Speed	−0.24	0.255	−0.12	0.584	−0.11	0.595	0.13	0.548

All data were checked for normality using Shapiro-Wilk’s test. Normal distributed data: Pearsson’s correlation (not underlined); Spearman’s correlation (underlined). Bold denotes significant correlations (P < 0.05). Italic denotes 0.10 > P > 0.05. PFO: Peak fat oxidation, FFM: Fat free mass, $\dot{\text{V}}$O_2peak_: Peak oxygen uptake.

## DISCUSSION

The present study examined the relationship between fat oxidation capacity and the ability to maintain physical performance during the final 30 minutes of football match play for elite male players. The main findings were threefold: a) fat oxidation capacity, when normalized to fat-free mass (PFO/FFM), was significantly negatively correlated with decrements in the number of decelerations in the final third of matches; b) $\dot{\text{V}}$O_2peak_ showed significant correlations with total match distance and submaximal running performance but was largely unrelated to intra-match performance decline; and c) PFO and Fatmax, did not correlate significantly with sprinting or high-speed running capacity in either absolute terms or intra-match changes. Collectively, these findings suggest that fat oxidation capacity may play a modest role in supporting repeated high-intensity accelerations and decelerations late in match play, whereas $\dot{\text{V}}$O_2peak_ was linked to total match running volume and distances covered both lower and higher speed zones.

The observed association between PFO/FFM and preserved capacity to perform decelerations indicate that a greater reliance on fat metabolism may mitigate fatigue-induced declines in neuromuscular performance. Although football is characterized by repeated highintensity bursts, more than 85% of match play occurs at submaximal intensities where aerobic pathways dominate [1, 8, 25]. Efficient fat oxidation during these phases may spare intramyofibrillar glycogen, thereby attenuating fatigue-related impairments in excitation– contraction coupling [10, 22] and thereby sustaining the ability to perform explosive movements. The association was only significant when PFO was expressed relative to fat-free mass (Figure 1), underscoring the relevance of body composition when interpreting metabolic traits in athletic populations [16]. However, the associations tended (P = 0.053) to be significant also when PFO were expressed in absolute terms.

Fat oxidation capacity, PFO, PFO/FFM and Fatmax, in elite football players, has been shown to be among the highest across sports [16]. Fat oxidation capacity in our population of professional football players was however more modest (PFO: 0.48 g/min; PFO/FFM: 6.95 mg/min/kg; Fatmax: 43%) compared with previous studies in sub-elite [17] and professional football players [9].

Unlike studies by Rømer and colleagues [17] and Randell and collegues [9], who conducted the fat oxidation capacity tests during pre-season, our tests were conducted within the competitive season. PFO is sensitive to recent training volume and intensity and increases in response to moderate-intensity endurance training [26] and during pre-season football training [17]. Players may have been tested during periods of cumulative fatigue or reduced aerobic loading (e.g. due to competitive congestion or tapering), which could have reduced mitochondrial enzymatic activity and lipid oxidation capacity. Thus, the difference in training status may partly explain differences to previous studies. Moreover, club nutritionists and players may pay more attention to a high carbohydrate intake during the competitive season compared to pre-season, leading to a more carbohydrate rich diet, which also may lower PFO, even though players were tested after ~6 hours fast, beyond which the length of the fast has been shown to have little effect on fat oxidation [27]. Nevertheless, in accordance with previous studies we observed large inter-individual variability [9, 16, 17]. While the present study included professional players from a football club across three seasons, the absence of individuals with particularly high PFO-values (> 0.8 g/min) and only two players with PFO/FFM higher than 10 mg/min/kg contributed to lower means. Moreover, it is possible that the inclusion criteria (match data availability and playing time) introduced unintentional selection bias favouring a more homogeneous metabolic profile. The inter-individual variability reinforces the notion that contextualizing PFO to individual physiological profiles, including lean mass and match demands, is crucial when exploring its functional relevance. In line with this, no significant correlation was observed between $\dot{\text{V}}$O_2peak_ and PFO, whether expressed in absolute terms or relative to fat-free mass. This suggests that aerobic capacity and maximal fat oxidation may reflect partly distinct physiological traits.

The current findings also contribute to the ongoing debate regarding the importance of $\dot{\text{V}}$O_2peak_ in relation to moderate- and high-intensity intermittent exercise performance [28–30]. In our study, $\dot{\text{V}}$O_2peak_ correlated strongly with total match distance (Figure 1) and distance covered at moderate intensities (Table 4), replicating previous reports linking aerobic capacity with general running load [28]. $\dot{\text{V}}$O_2peak_ showed, however, limited relevance for within-match performance decrements, except for a single association with jogging distance in the final 30 minutes (Table 3).

No laboratory-derived variable was associated with sprint distance, number of sprints, or top speed, neither across the full match nor in the final 30 minutes. These match performance metrics are highly linked to neuromuscular power and anaerobic capacity [1, 8, 30]. It is therefore unsurprising that neither fat oxidation nor $\dot{\text{V}}$O_2peak_ accounted for variability in these metrics. A relationship between fat oxidation and the fatigue resistance has been shown in semi-professional players, as a study by Mohr, Thomassen [31] found that total distance covered, distance deficit from first to second half, distance covered with high-intensity running (> 14 km/h) as well as high-intensity running in the final 15 minutes of the match were correlated with beta-hydroxyacyl-CoAdehydrogenase (HAD) maximal activity. However, we could not confirm this association in the current study.

The lack of association between Fatmax and any match variable further suggests that the absolute capacity for fat oxidation, rather than the relative intensity at which it peaks, may be more relevant for maintaining performance capacity in the latter part of a match. Fatmax is known to vary substantially between individuals and is influenced by training background, nutrition, and testing protocol [20]. In contrast, PFO expressed relative to fat-free mass (PFO/FFM) may offer a more physiologically valid and comparable metric across athletes, as it reflects the fat oxidation capacity per unit of metabolically active tissue. This normalization reduces the influence of body size and may better represent underlying muscular oxidative adaptations [20].

From a practical perspective, the present findings offer a cautious support of the relevance of fat oxidation capacity as a supporting factor for late-game performance. Although significant, the associations between fat oxidation and match performance explained less than 18% of the variance, highlighting that fat oxidation capacity plays only a modest role relative to other determinants of performance. While the observed correlations were limited, the ability to maintain high-intensity actions such as decelerations may partly be supported by the glycogen sparring effect of increased fat oxidation capacity. Training strategies aimed at optimizing fat metabolism, including submaximal aerobic training or nutritional periodization, may enhance players’ ability to maintain performance. However, given the observed moderate effect sizes, few correlations, and marked inter-individual variation, implementation of such strategies should be guided by individual profiling. While metabolic flexibility may benefit fatigue resistance, it must be supported by a robust glycolytic system to meet the full range of demands met in elite football.

Several limitations must be acknowledged. First, while reasonable for elite sport settings, the sample size limits generalizability and may have reduced the power to detect smaller associations. Second, the time interval between laboratory testing and match performance varied, potentially introducing noise. Third, only one match per player was included, and tactical or positional influences were not statistically controlled. Match variability has been shown to be rather large especially for the higher speed zones and accelerations and decelerations [32–34], which may have impacted the possibility to detect relevant associations between laboratory test results and match performance metrics. Furthermore, positional differences in match activity profiles likely influence energy metabolism and substrate utilization during matches. Due to the modest sample size, we were not able to conduct reliable position-specific sub-analyses, which should be considered in future work. Likewise, the nutritional context before matches, particularly pre-match meals, albeit served at the same time and with the same content for all players, and carbohydrate availability, may have influenced substrate utilization and thereby the strength of associations between laboratory-derived fat oxidation capacity and in-game performance [35]. These contextual factors are inherent to studies in elite sport environments and should be acknowledged when interpreting the findings in the current study.

The present findings suggest that enhancing fat oxidation capacity relative to fat-free mass may help players sustain repeated deceleration efforts in the final stages of elite football matches. Conditioning programmes should therefore integrate aerobic training and nutritional periodization to improve metabolic flexibility, while also incorporating neuromuscular-focused training to maintain high-intensity performance under fatigue. Importantly, such strategies should be tailored to individual physiological profiles and supported by regular monitoring of metabolic characteristics.

## CONCLUSIONS

In conclusion, the present findings support a modest but significant role for fat oxidation capacity in sustaining specific aspects of physical performance during male elite football matches, particularly in relation to repeated deceleration efforts late in games. $\dot{\text{V}}$O_2peak_ was shown to be correlated to overall running performance but appears less informative for fatigue resistance. Together, these results suggest that fat oxidation profiles, particularly when normalized to fat-free mass, may serve as an additional metabolic marker to inform individualized conditioning strategies in professional male football.
